# *In vitro* Evaluation of Programmed Cell Death in the Immune System of Pacific Oyster *Crassostrea gigas* by the Effect of Marine Toxins

**DOI:** 10.3389/fimmu.2021.634497

**Published:** 2021-04-01

**Authors:** Norma Estrada, Erick J. Núñez-Vázquez, Alejandra Palacios, Felipe Ascencio, Laura Guzmán-Villanueva, Rubén G. Contreras

**Affiliations:** ^1^Programa Cátedras CONACyT (Consejo Nacional de Ciencia y Tecnología), Centro de Investigaciones Biológicas del Noroeste, S.C. (CIBNOR), La Paz, Mexico; ^2^Laboratorio de Toxinas Marinas y Aminoácidos, Centro de Investigaciones Biológicas del Noroeste, S.C. (CIBNOR), La Paz, Mexico; ^3^Laboratorio de Patogénesis Microbiana, Centro de Investigaciones Biológicas del Noroeste, S.C. (CIBNOR), La Paz, Mexico; ^4^Departamento de Fisiología, Biofísica y Neurociencias, Centro de Investigación y de Estudios Avanzados del IPN (CINVESTAV), Mexico City, Mexico

**Keywords:** programmed cell death, marine toxins, apoptosis, pyroptosis-like, bivalve mollusk, *Crassostrea gigas*

## Abstract

Programmed cell death (PCD) is an essential process for the immune system's development and homeostasis, enabling the remotion of infected or unnecessary cells. There are several PCD's types, depending on the molecular mechanisms, such as non-inflammatory or pro-inflammatory. Hemocytes are the main component of cellular immunity in bivalve mollusks. Numerous infectious microorganisms produce toxins that impair hemocytes functions, but there is little knowledge on the role of PCD in these cells. This study aims to evaluate *in vitro* whether marine toxins induce a particular type of PCD in hemocytes of the bivalve mollusk *Crassostrea gigas* during 4 h at 25°C. Hemocytes were incubated with two types of marine toxins: non-proteinaceous toxins from microalgae (saxitoxin, STX; gonyautoxins 2 and 3, GTX2/3; okadaic acid/dynophysistoxin-1, OA/DTX-1; brevetoxins 2 and 3, PbTx-2,-3; brevetoxin 2, PbTx-2), and proteinaceous extracts from bacteria (*Vibrio parahaemolyticus*, Vp; *V. campbellii*, Vc). Also, we used the apoptosis inducers, staurosporine (STP), and camptothecin (CPT). STP, CPT, STX, and GTX 2/3, provoked high hemocyte mortality characterized by apoptosis hallmarks such as phosphatidylserine translocation into the outer leaflet of the cell membrane, exacerbated chromatin condensation, DNA oligonucleosomal fragments, and variation in gene expression levels of apoptotic caspases 2, 3, 7, and 8. The mixture of PbTx-2,-3 also showed many apoptosis features; however, they did not show apoptotic DNA oligonucleosomal fragments. Likewise, PbTx-2, OA/DTX-1, and proteinaceous extracts from bacteria Vp, and Vc, induced a minor degree of cell death with high gene expression of the pro-inflammatory initiator caspase-1, which could indicate a process of pyroptosis-like PCD. Hemocytes could carry out both PCD types simultaneously. Therefore, marine toxins trigger PCD's signaling pathways in *C. gigas* hemocytes, depending on the toxin's nature, which appears to be highly conserved both structurally and functionally.

## Introduction

Pacific oyster *Crassostrea gigas* (Thunberg, 1793) (Bivalvia, Mollusk) shows the highest aquaculture production in the world and is one of the best-studied bivalve mollusks (1, 2). These benthic invertebrates filter high volumes of water through their gills and accumulate pathogenic microbes and environmental toxins that continuously challenge their normal functions (3–5). To cope with this challenge, these animals have developed effective systems to detect and discriminate beneficial microorganisms from potentially harmful and pathogenic ones and are capable of keeping infections under control (6–8). Bivalve's innate immunity consists of humoral components of hemolymph (agglutinins, lysosomal enzymes, opsonizing molecules, and antimicrobial peptides) and the cellular defense that the hemocytes perform (9). Hemocytes represent the first line of internal defense against parasites, pathogens, and non-self-materials in bivalve mollusks and play a significant role in the immune system homeostasis and disease prevention. They are capable of phagocytosis, encapsulation, and enzymatic digestion (9–14), and participate in other processes, such as wound and shell repair, nutrient digestion, transport, and excretion (10, 15). Injury, toxic substances, or invasion by pathogenic microorganisms activates internal hemolymph factors such as hormones, cytokines, and other humoral factors that regulate hemocyte's function and migration to promote localized responses (14, 16, 17).

Hemocyte death is a naturally occurring phenomenon in bivalve mollusks due to internal and external stimuli, as in other multicellular organisms. There are two types of cell death: 1) programmed/regulated cell death (PCD), most commonly known as apoptosis, but including also autophagy, necroptosis, pyroptosis, and 2) necrosis, a kind of accidental cell death due to non-physiological states such as infection or injury (18, 19). PCD is a natural part of the animal cell cycle and an essential factor in animal disease progression. In a healthy animal, PCD occurs when a cell is damaged, infected, senescent, or otherwise of little use to the animal and plays crucial roles in immune system homeostasis and function, defense against parasite and pathogens, and self/non-self recognition (20–24). Hemocytes' enhanced PCD could conceivably create immunosuppression that in turn would reduce disease's resistance, increase opportunistic infections, and decline mollusk's population (25–31).

PCD involves activating a family of cysteine proteases called caspases (32, 33), that can be pro-apoptotic or pro-inflammatory. The pro-apoptotic subfamily includes the initiator caspases -2, -8, -9, and -10 that respond to the apoptotic signals and cleave and activate the effector caspases -3, -6, and -7, which in turn cleave target proteins to orchestrate apoptotic cell death (21, 28, 31, 32, 34). Apoptotic cell death is an immunologically silent death that does not induce inflammation but allows the orderly degradation and recycling of cellular components. The pro-inflammatory caspases -1, -4, -5, and -11 play a significant role in innate immune responses by inducing pyroptosis, an inflammatory cell death that clear infections by removing pathogen replication niches and releasing pro-inflammatory cytokines and danger signals (21, 34–38). Caspases induce profound changes in cells, including the hallmarks of apoptosis: phosphatidylserine (PS) translocation from the cytosolic to the exoplasmic leaflet of the plasma membrane, cell shrinkage and blebbing, chromatin condensation, and DNA nuclear fragmentation (39–41). Pyroptosis also exhibits PS translocation, resulting from plasma membrane rupture, nuclear condensation, and DNA cleavage, but nuclear integrity is maintained (42–45). Table 1 summarizes the features of apoptosis and pyroptosis.

**Table 1:** Generalized features of apoptosis and pyroptosis.

	**Features**	**Apoptosis**	**Pyroptosis**
Outcome	Evolutionary conserved	Yes	Yes
	Programmed	Yes	Yes
	Regulated process	Yes	Yes
	Inflammatory	No	Yes
	Signaling pathway	Specific	Specific
	Activated by	Intrinsic or extrinsic	PAMPs and DAMPs
Intermediate signaling	Mitochondrial disfunction	Yes	Yes
	Cytochrome-c release	Yes	No
	Caspase-1	No	Yes
	Caspase-2	Yes	No
	Caspase-3	Yes	Yes^*^
	Caspase-7	Yes	No
	Caspase-8	Yes	Yes^*^
Phenotype	Membrane intact	Yes	No
	Pore formation	No	Yes
	Membrane blebbing	Yes	No
	Cell lysis	No	Yes
	Cell swelling	No	Yes
	PS exposure	Yes	Yes
	Chromatin condensation	Yes	Yes
	DNA fragmentation	Yes	Yes
	DNA laddering	Yes	No

* *Variable according to cell type*.

A wide variety of pathogenic microorganisms like viruses, bacteria, protozoan, and microalgae, cause mollusk hemocyte cell death, either as a consequence of infecting host cells or producing toxic products. The study of these effects has raised interest to solve the economic, ecologic, and health challenges of mollusk's aquaculture (5, 25, 30, 46–62). The objective of this study was to demonstrate whether marine toxins induce PCD in hemocytes. We exposed *C. gigas* hemocytes *in vitro* to non-proteinaceous microalgae marine toxins saxitoxin (STX), gonyautoxins 2 and 3 (GTX2/3), brevetoxins 2 and 3 (PbTx-2,-3), brevetoxin 2 (PbTx-2), okadaic acid/dynophysistoxin 1 (OA/DTX-1), as well as proteinaceous toxins from the bacteria *Vibrio parahaemolyticus* (Vp) and *V. campbellii* (Vc). Little is known, about how PCD processes regulates bivalve's immune defenses, and if the pathogens or xenobiotics induce hemocyte's PCD to unbalance cellular homeostasis toward higher mortality.

## Materials and Methods

### Source of Oysters

*Crassostrea gigas* (Thunberg, 1793) oysters (11 ± 1.2 cm) cultivated in suspended cages at Rancho Bueno, Mexico (24°32 N, 111°42 W), were collected and transported to CIBNOR. The specimens were placed in 40 L plastic tanks containing filtered (1 μm) seawater (35 psu) pumped directly from the sea. The water was maintained with constant aeration through air stones. The water was replaced every 2 days. During acclimation (10 days), oysters were fed a mixture of microalgae (*Chaetoceros calcitrans, C. muelleri*, and *Isochrysis galbana*; 1:1:1) obtained at CIBNOR. *C. calcitrans* (CHCAL-7) and *C. muelleri* (CHM-8) were cultured in 20-L plastic bags in F/2 growth medium at 22°C under constant illumination at a salinity of 32 PSU. *I. galbana* (ISG-1) was grown in MA-F/2 medium at the same temperature, salinity, and volume under continuous illumination, and were harvested in the stationary growth phase.

### Toxins and Apoptosis Inducers

#### Obtention, Extraction, and Quantification of Marine Toxins

##### Non-proteinaceous Toxins

###### Paralyzing Shellfish Toxins.

Saxitoxin (STX) FDA Reference Standard Saxitoxin was obtained from the US National Institute of Standards and Technology (NIST, RM 8642). The saxitoxin dihydrochloride concentration is nominally 100 μg mL^−1^ in a solution of 80% acidified water (pH 3.5) and 20% ethanol (volume fractions) and provided by Marine Toxins and Amino acids Laboratory from CIBNOR. Gonyautoxins epimers 2 and 3 (GTX 2/3) were obtained according to Estrada et al. (58) from cultured dinoflagellate *G. catenatum* (Strain GCQM-2) (https://www.cibnor.gob.mx/investigacion/colecciones-biologicas/codimar). Identity and quantification of STX and GTX 2/3 were subjected to HPLC analysis, using the post-column oxidative fluorescence method (63, 64), and the biological activity was performed by mouse bioassay (MBA) according to AOAC (65) standards.

###### Diarrheic Shellfish Toxins.

A mixture of okadaic acid (OA) and dinophysistoxin 1 (DTX-1) were obtained from cultured of the dinoflagellate *Prorocentrum lima* (Strain PRL1) isolated from the Gulf of California (66), and provided by Marine Toxins and Amino acids Laboratory from CIBNOR. The cells were cultured in f/2+Se medium (67, 68) with filtered (0.45 μm) seawater and grown in monoalgal cultures in 500 mL Erlenmeyer flasks for 12-h light:12-h dark photocycle at 25°C under 70 W fluorescent lamps and anaerobic conditions. The concentration of OA/DTX-1 in the extract semi-purified (69) was calculated as log Mouse Unit (MU) = 2.6 log (1 + t−1); MU = 4 μg of OA (70). The DST content (OA + DTX-1) was determined by LC–MS/MS method (71).

###### Neurotoxic Shellfish Toxins.

The brevetoxin 2 (PbTx-2) extract and the mixture of brevetoxin 2 and 3 (PbTx-2,−3) were obtained by cultivation of the dinoflagellate *Karenia brevis* (Strain Kb-3) originally isolated from the Gulf of Mexico and donated to CIBNOR by Dr. Tracy Villareal from the University of Austin, Texas, USA. The cells were cultured in GSe medium (72) with filtered (0.45 μm) seawater and grown in monoalgal cultures in 2.8 L Fernbach flasks for 12-h light:12-h dark photocycle, at 25°C under 70 W fluorescent lamps and anaerobic conditions. Extraction and semi-purification of PbTx2 and PbTx-2,-3 were performed and provided by Marine Toxins and Amino acids Laboratory from CIBNOR (73). PbTxs were identified and measured by LC-MS/MS (pers. comm. Dr. Andrew Turner, CEFAS, United Kingdom), and MBA measured biological activity according to the American Public Health Association (74).

We made aliquots for these non-proteinaceous marine toxins, and the solvents were removed by evaporation to dryness *in vacuo* and stored at −80°C. Before experiments toxins were suspended in 1% dimethyl-sulfoxide (DMSO), diluted in 0.22 μm sterile saline solution (Cs PiSA NaCl 0.9%, pH 7.2), prepared immediately before use for the desired working concentration.

##### Proteinaceous Toxins

*Crude Extracts of Bacteria Vibrio parahaemolyticus and V. campbellii. V. parahaemolyticus* (Strain VpM) and *V. campbellii* (VcA1) were isolated from white-leg shrimp (*Litopenaeus vannamei*) and Pacific oyster (*C. gigas*), respectively, with signs of illness and growth in Luria-Bertani agar (LB), and then transferred to Miller's LB Broth (37°C). Ten liters of each strain (1 × 10^9^ cell mL^−1^) was centrifuged at 600 × g, 10 min at 4°C, and freeze. To confirm the strains' identity, we extracted DNA by the organic extraction method (75), and DNA was resuspended by the addition of TE Buffer 10 mM pH 8.0. DNA purity and concentration were estimated using the Nanodrop 1000 spectrophotometer (Thermo Fisher Scientific). DNA integrity was visualized in 1.5% agarose gel electrophoresis, under UVP Biodoc-It 2 imaging system (Analytik Jena), stained with the fluorescent dye GelRed^®^ Nucleic Acid Gel Stain (Biotium 41003). Endpoint PCR was performed to identify the bacterial species and identify some virulence factors in the strains. The primers used are shown in Supplementary Table 1. PCR reactions were performed with GoTaq^®^ Flexi DNA Polymerase (Promega M829) according to the manufacturer's instructions. The PCR products were electrophoresed and sequenced by Genewiz (South Plainfield, NJ, USA). The sequences were analyzed by Blast-NCBI (https://blast.ncbi.nlm.nih.gov/Blast.cgi). Supplementary Table 2 shows the genes identified in bacterial strains. To obtain the protein crude extract, centrifuged bacteria were re-suspended in filtered (0.2 μm) sterile saline solution (CS PiSA NaCl 0.9%, pH 7.2), with 1X protease inhibitor (Sigma P1860), and 0.5% Triton X-100. Cells were homogenized with glass beads (300 μm) in a vortex, and the extract was centrifuged at 1,200 × g, 15 min, 4°C. Protein was determined by BCA Protein Assay Kit (bicinchoninic acid, 23227, Thermo Fisher Scientific) according to the manufacturer's instructions with bovine albumin as a standard.

##### Commercial Apoptosis Inducers

Camptothecin (CPT, C-9911, Sigma–Aldrich, St. Louis, MO) was dissolved in DMSO and made into a stock 1 mM solution. Staurosporine (STP, 81590, Cayman Chemical, Ann Harbor, MI) was dissolved in DMSO and made into a 1 mM stock solution.

### Hemolymph Extraction

Oyster shells were surface-cleaned with 70% ethanol, and hemolymph (4–5 pools of 10–30 animals) extracted from the adductor muscle using a 26-gauge hypodermic needle making a small hole in the valves of the animals close to the muscle, and immediately put on ice. No oyster was subjected to more than one sampling. Immediately after hemolymph collection, the total number of hemocytes was determined using TC20™ Automated Cell Counter (BioRad). The mean cell concentration for the hemocyte oyster population was 1.9 ± 0.5 × 10^6^ cell mL^−1^. When necessary, hemocytes' concentration in the solution was adjusted by adding centrifuged hemolymph (600 × g, 10 min, 4°C) without hemocytes.

#### Biological Activity of Toxins and Commercial Apoptosis Inducers

To assess toxins and commercial apoptosis inducer's biological activity, we carried out a hemocyte viability test with resazurin sodium salt (C-62758-13-8, Sigma-Aldrich). Each hemocyte pooled subgroup was subdivided into three aliquots (each 100 μL at densities of 1 × 10^6^ cell mL^−1^) for each treatment. Aliquots were placed in a 96-well sterile microplate and allowed to attach and spread for 1 h at 25°C. Samples were exposed to three different concentrations of STP, CPT, toxins, negative control (hemocytes exposed to sterile saline Cs PiSA NaCl 0.9%, pH 7.2), or positive control (hemocytes exposed to 10 mM HCl pH 2). Not more than 10 μL of toxins or apoptosis inducer's working concentration were added for every 100 mL of hemolymph to obtain the final concentrations studied. Control tests with DMSO were assayed and showed no effect on cell viability (data not shown). Hemocytes were incubated in the dark for 4 h at 25°C, in a humid chamber in triplicate. Preview studies have shown that incubation for 4–6 h is enough to induce apoptosis in bivalve hemocytes exposed to marine toxins such as PST and DST (56, 58). Following the incubation time, cell viability was measured with the resazurin reduction cell viability assay (76). From this experiment, final concentrations close to 50% mortality were chosen for the rest of the tests (Table 2).

**Table 2:** Final concentration of apoptosis inducers or marine toxins used for the experiments.

**Apoptosis inducers or marine toxins**	**Concentration**
STP	1.5 μg mL^−1^
CPT	1.2 μg mL^−1^
STX	5 μg STX *eq* mL^−1^
GTX 2/3	1.25 μg STX *eq* mL^−1^
OA/DTX-1	3 μg AO *eq* mL^−1^
PbTx2	5 μg PbTx *eq* mL^−1^
PbTx-2,3	4 μg PbTx *eq* mL^−1^
Vp	6.25 μg protein mL^−1^
Vc	5 μg protein mL^−1^

*CPT, Camptothecin; STP, Staurosporine; STX, Saxitoxin; GTX, Gonyautoxin; OA, Okadaic acid; DTX, Dynophysistoxin; PbTx, Brevetoxin; Vp, Vibrio parahaemolyticus extract; Vc, V. campbellii extract*.

### Neutral Comet Assay

Neutral comet assay detects the breakage of double-stranded DNA (58, 77). Aliquots of 150 μL of hemolymph, with a cellular concentration of 1.5 × 10^6^ cells mL^−1^, were placed in Eppendorf tubes. The cells were exposed to toxins or apoptosis inducers, according to Table 2. Hemocytes were incubated for 4·h at 25°C, in a dark, humid chamber in triplicate. Hemocytes were harvested using 0.25% trypsin in filtered (0.2 μm) sterile seawater (pH 7, 33 PSU) and washed and resuspended in the same sterile volume seawater. The suspension was added to 0.75% low-temperature melting agarose at a ratio of 1:10 (v/v) and spread on glass slides that were pre-coated with 0.7% regular agarose and then air-dried. Slides with double-layered agarose were submerged in pre-cooled lysis solution (154 mM NaC1, 10 mM Tris, 10 mM EDTA, and 0.5% SLS at pH 10) at 4°C for 30 min, washed briefly to remove detergent and salt, and electrophoresed at ~7 V cm^−1^ for 3 min in TBE solution (40 mM Tris-boric acid, 2 mM EDTA at pH 8.3) and then stained for 10 min with propidium iodide (PI; 10 μg mL^−1^). DNA damage was quantified by measuring displacement between the nucleus's genetic material (comet head) and the resulting tail, according to Estrada et al. (58); the comet tail's intensity, relative to the head, reflects the number of DNA breaks.

### Annexin V Assay

To identify PS exposure on the outer leaflet of the plasma membrane, we used the Annexin V–FITC apoptosis kit (BioVision, K101). Each of the pooled subgroups was subdivided into three aliquots (aliquots of 200 μL at densities of 1 × 10^6^ cell mL^−1^) for each treatment, put directly on a coverslip and allowed to attach and spread for 1 h at room temperature. The cells were exposed to toxins or apoptosis inducers, according to Table 2. Hemocytes were incubated for 4 h at 25°C, in a dark, humid chamber in triplicate. Following exposure to toxins or apoptosis inducers, hemocytes were washed with filtered (0.2 μm) sterile seawater (pH 7, 33 PSU). The hemocytes were processed with Annexin-V according to the manufacturer's instructions. Following incubation, the coverslip was inverted on a glass slide and observed under a phase-contrast microscope (Nikon Eclipse Ni-U) coupled with fluorescence for characterization using a dual filter set for FITC and rhodamine. At least 100 cells were counted in each sample. Categories were assigned based on the total number of hemocytes counted. Viable cells are stained for annexin V (FITC green) but not for propidium iodide (PI, red). Cells in early PCD are stained by FITC-annexin V but not by PI. Late PCD or already dead cells are stained both by FITC-annexin V and PI, or only by PI, respectively.

### Chromatin Condensation

We used adherent cells stained with 4′,6-Diamidino-2-Phenylindole (DAPI, ThermoFisher D1306) to evaluate chromatin's condensation. Hemocytes (200 μL at densities of 1 × 10^6^ cell mL^−1^) were allowed to attach and spread onto a glass coverslip for one h at room temperature. Then they were exposed to toxins or apoptosis inducers for 4 h at 25°C, washed with filtered (0.2 μm) sterile seawater (pH 7, 33 PSU), and fixed with methanol. Fixed hemocytes were immersed in PBS buffer (137 mM NaCl, 2 mM KCl, 10 mM Na_2_HPO_4_, 1.8 mM KH_2_PO_4_, pH 7.2) for 5 min and then treated with a DAPI solution in PBS (1:1,500) for 5 min. Hemocytes were washed with PBS, and the coverslip was inverted and mounted on a glass slide and observed under a fluorescence microscope (365 nm) (Nikon Eclipse Ni-U). The percentage of nuclei with chromatin condensation was estimated by examining 200 cells per sample. Cells with intact DNA show weak fluorescence signals; in contrast, cells with condensed chromatin exhibit stronger fluorescence when observed under a fluorescence microscope. Also, condensed chromatin could be kept in the periphery of the nuclei or small fragments dispersed.

### DNA Fragmentation Assay

Aliquots of 2 mL of hemolymph, with a cellular concentration of 2 × 10^6^ cells mL^−1^, were placed in Eppendorf tubes in triplicate. The cells were exposed to toxins or apoptosis inducers, according to Table 2. Each hemocyte triplicate was pooled to extract DNA with the Apoptotic DNA-Ladder Kit (Merck 11835246001), according to the manufacturer's instructions. DNA was visualized in 2% agarose gel electrophoresis, under UVP Biodoc-It 2 imaging system (Analytik Jena), stained with the fluorescent dye GelRed^®^ Nucleic Acid Gel Stain (Biotium 41003).

### Gene Expression Analysis by Quantitative Real-Time PCR

#### RNA Extraction and cDNA Synthesis

To perform RT-qPCR analysis, after hemolymph extraction, 2 mL of hemolymph with a cellular concentration of 1.5 × 10^6^ cells mL^−1^ were placed in Eppendorf tubes and exposed to the desired final concentration of toxins or apoptosis inducers according to Table 2. Hemocytes were incubated for 4 h at 25°C, in a humidity chamber in triplicate. Following incubation hemocytes were centrifuged 600 × g, 15 min, 4°C, and washed in filtered (0.2 μm) sterile seawater (pH 7, 33 PSU). The hemocyte pellet obtained was stored to −80°C for further analyses. For total RNA extraction pooled hemolymph samples were lysed in 1 mL of FastRNA^®^ Pro Green Kit solution (MP Biomedicals) and processed according to the manufacturer's instructions. The extracted RNA concentration was measured by spectrophotometer (Nanodrop 1000^®^ Thermo Scientific) at 260 nm. The purity of RNA was determined as the 260/280 nm ratio with acceptable values > 1.8. RNA concentration was estimated using the Nanodrop 1000 spectrophotometer (Thermo Fisher Scientific). A total of 1 μg total RNA was treated with 1 U DNase I (SIGMA AMPD1) for 2 h at 37°C and then heat-inactivated at 65°C for 10 min before reverse transcription to eliminate genomic DNA contamination. The integrity of total RNA was analyzed by 1% agarose gel electrophoresis under UVP Biodoc-It 2 imaging system (Analytik Jena), stained with the fluorescent dye GelRed^®^ Nucleic Acid Gel Stain (Biotium 41003). A sample of 2.5 μg RNA was used to synthesize cDNA from each pooled sample using an oligo dT and Superscript III first-strand synthesis system for RT-PCR kit (Invitrogen, USA 11904018), according to the manufacturer's instructions. The resulting cDNA was stored at −80°C until use. cDNA synthesis was confirmed by endpoint PCR amplification of the beta actin gene (Forward 5′-CCACACCCGTAAGGGAAAG-3′; Reverse 5′-GGTTACCACCACCATGAGG-3′) with GoTaq^®^ Flexi DNA Polymerase (Promega M829), and PCR products were electrophoresed in 1% agarose gel with GelRed^®^ Nucleic Acid Gel Stain (Biotium 41003).

#### Quantitative Real-Time PCR

cDNAs were used for qPCR analysis to determine the relative expression of mRNA coding five caspases (caspase 1, 2, 3, 7, and 8) and two endogenous controls (RPL7 and RPL36). Primers were obtained from preview reports (Supplementary Table 1), and the primers were ordered from T4 Oligo (Irapuato, Gto, Mexico). Primers efficiency was tested using the standard curve method. For this purpose, a serial dilution (1:5, 1:10, 1:20, 1:40, 1:80) was made from a single cDNA sample consisting of a pool of all cDNAs different treatments (0.5 μg μL^−1^). Only primers that showed efficiencies between 1.8 and 2.2 were used. The qPCR analysis was performed in tube strips in triplicate using the Rotor-Gene Q (Quiagen TM) with a total reaction volume of 10 μL. Each reaction had 5 μL of 2X SsoFast™EvaGreen^®^Supermix (Bio-Rad, Hercules, CA, 1725201), 0.3 mM of each primer, and 1 μL of each diluted cDNA (100 ng μL^−1^). Amplification conditions were enzyme activation at 95°C for 1 min, followed by 40 cycles of denaturation 10 s at 95°C and annealing/extension 30 s at 59°C. The qPCR product's specificity was analyzed by a dissociation curve performed after amplification (65–95°C continuous-time), observing a single peak at the expected Tm. Relative quantification of the expression of the analyzed genes was calculated using REST 2009 (Relative Expression Software Tool) software v2.0.13 with RG mode (http://www.qiagen.com/rest), using the pair-wise fixed randomization test (78). Normalization using the housekeeping genes RPL7 and RPL36 were used to identify the expression levels of the caspase's genes. Using the take-off values obtained from Rotor-Gene Q, the program performed 3,000 iterations to determine whether there are significant differences between samples and controls while considering issues of reaction efficiency and reference gene normalization. This program's expression values are a ratio such that values above 1 denote an upregulation of gene expression in the treated group while values <1 indicate a downregulation (*P < 0.05; **P < 0.01). Expression variation for each gene is visualized in a whisker-box plot.

### Statistical

For all experiments, means and SD were calculated, and results are expressed as the means ± SD of three independent experiments, except for RT-qPCR analysis as mentioned above. Comparisons between control and treatments were assessed with Student's t distribution or Wilcoxon test, according to results obtained with Shapiro-Wilk (distributions normality) and Fisher (homoscedasticity) tests. Statistical significance was set at *P* < 0.05. All analyses were performed using the SPSS for Windows statistical package (version 16.0).

## Results

### Cytotoxicity of Hemocytes

**Figure 1:**
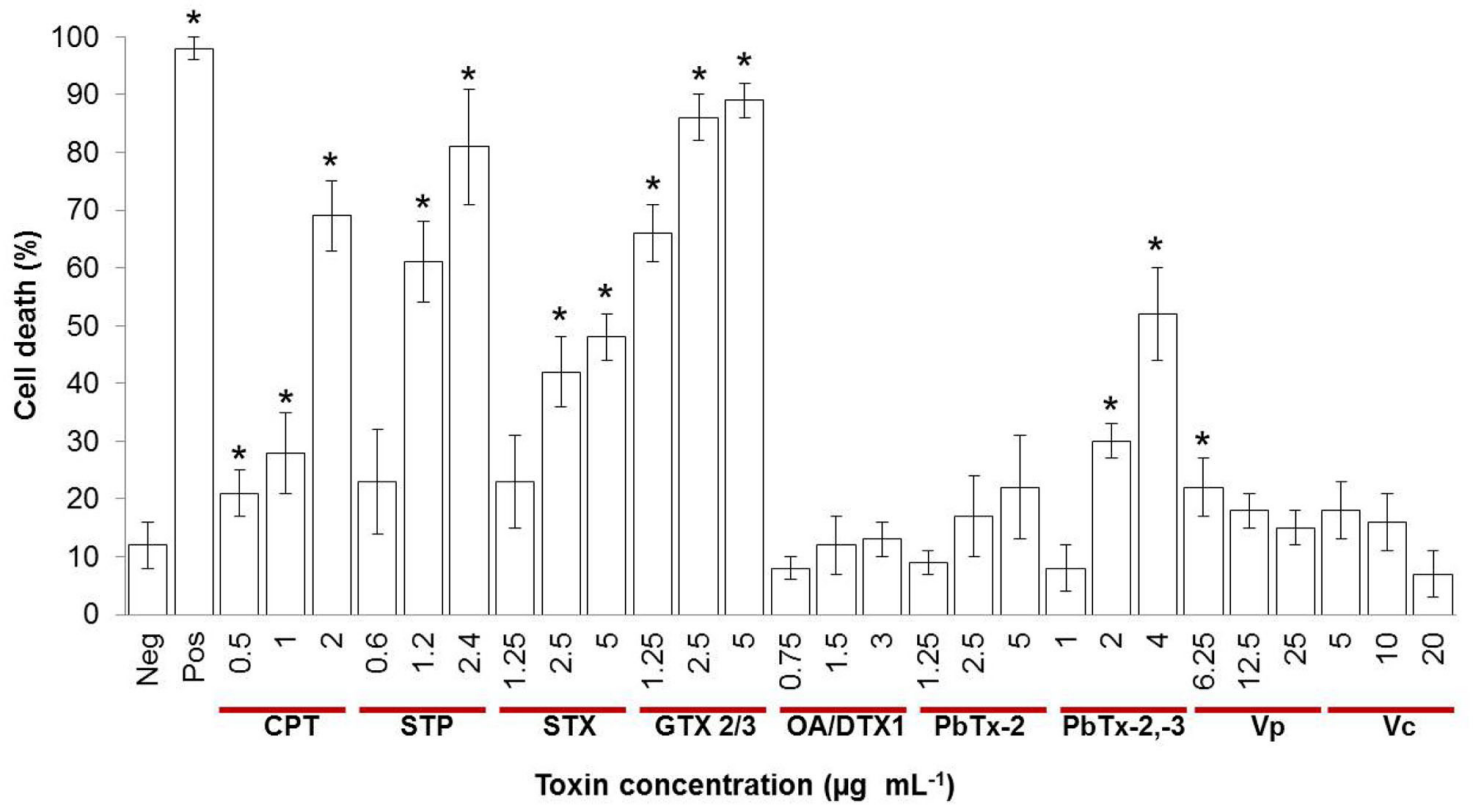
Percentage of cell death evaluated through the resazurin assay in hemocytes treated with different concentrations of inducers of apoptosis or marine toxins during 4 h at 25°C. Hemolymph from 10 to 30 oysters was pooled to have a total of 1.5 × 10^6^ cells mL^−1^. Bars represent mean ± standard deviation of two independent experiments. *P < 0.05. Neg, negative control; Pos, positive control; CPT, camptothecin; STP, staurosporine; STX, saxitoxin; GTX, gonyautoxin; OA, okadaic acid; DTX, dynophysistoxin; PbTx, brevetoxin; Vp, *Vibrio parahaemolyticus* extract; Vc, *V. campbellii* extract.

To investigate if marine toxins cause hemocyte toxicity, we measured cell viability after 4 h of exposure to proteinaceous and non-proteinaceous toxins and apoptosis inducers (Figure 1). Control cells in sterile saline NaCl, 0.9% (Neg) showed negligible cell viability changes, while hemocytes treated with 10 mM HCl pH 2 (Pos) showed the expected cell death of 97%. Staurosporine (STP), camptothecin (CPT), saxitoxin (STX), gonyautoxin epimers 2 and 3 (GTX2/3), and the mixture of brevetoxins 2 and 3 (PbTx-2,-3) provoked a dose-dependent effect, with hemocyte mortality above 50% for the highest concentrations. The mixture of okadaic acid and dinophysistoxin 1 (AO/DTX-1) was not toxic at the concentration range tested, and brevetoxin 2 (PbTx-2) exerted minor toxicity only at 5 μg mL^−1^. Crude extracts of *Vibrio parahaemolyticus* (Vp) and *V. campbellii* (Vc) increased cell death only at a marginal level of 5–10%, at 6.25 and 5 μg mL^−1^, respectively. These results demonstrate that marine toxins induce hemocytes' cell death.

### Phosphatidylserine Translocation

**Figure 2:**
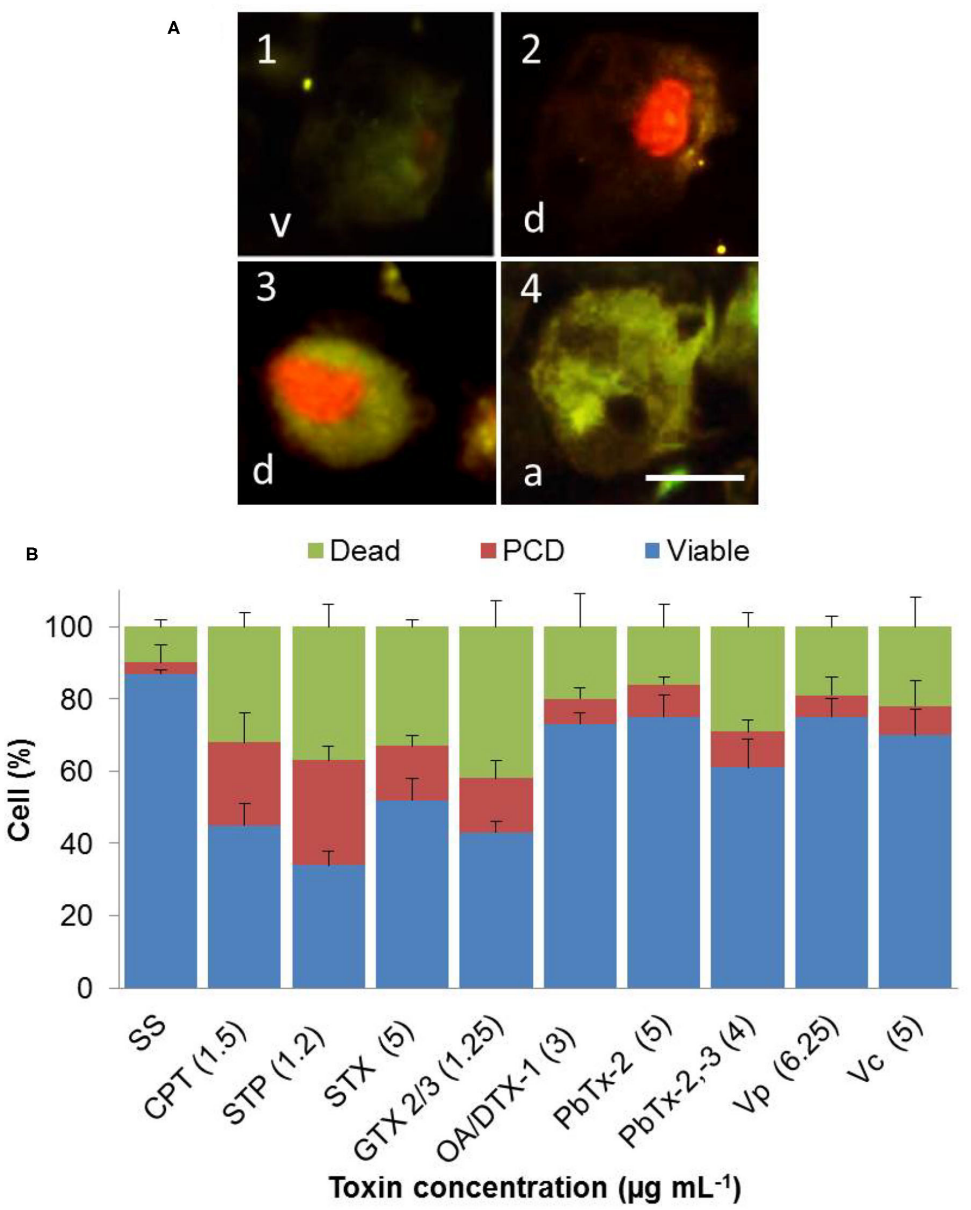
*In vitro* phosphatidylserine translocation to the extracellular leaflet in hemocytes exposed to apoptosis inducers or marine toxins during 4 h at 25°C. (A1) Hemocytes observed by fluorescence, to detect viable or no measurable programmed cell death (PCD, green and red staining negative), (A4) PCD cells (green, annexin V-bound), and (A2 and A3) cells in end stage of PCD and dead (red, propidium iodide stained cells, and green annexin V-bound cells). **(B)** The graph shows percentages of different stages of cells of **(A)**. Results are expressed as the mean ± standard deviation. A, annexin V positive; d, dead; v, viable. Scale bar = 5 μm. SS, saline solution; CPT, camptothecin; STP, staurosporine; STX, saxitoxin; GTX, gonyautoxin; OA, okadaic acid; DTX, dynophysistoxin; PbTx, brevetoxin; Vp, *Vibrio parahaemolyticus* extract; Vc, *V. campbellii* extract.

We used Annexin V to identify PS's translocation from the cytoplasmic to the exoplasmic leaflet of the hemocyte plasma membrane, through the binding of fluorescent annexin V at 4 h post challenged. Figure 2A showed hemocytes observed by fluorescence microscopy to detect viable or no measurable PCD cells (green and red staining negative, **v**), PCD cells (green, anexin V-bound, **a**), and cells in end stage of PCD and dead (red, propidium iodide stained cells, and green anexin V-bound cells, **d**). We measured the percentage of hemocytes at each of these different stages, after 4 h of incubation in media with marine toxins. We choose the lowest concentration of marine toxins that provoked 50% of hemocyte death, or the concentration that caused the highest response, to perform this experiment (Table 2). We observed 2% of PCD (red column) in sterile saline solution NaCl 0.9% (Figure 2B, SS). Incubation with CPT, STP, STX, and epimers GTX 2/3, increased PCD by ~15–30% (Figure 2B), demonstrating that these toxins induce PCD.

### Breakage of Double-Stranded DNA and Chromatin Condensation

**Figure 3:**
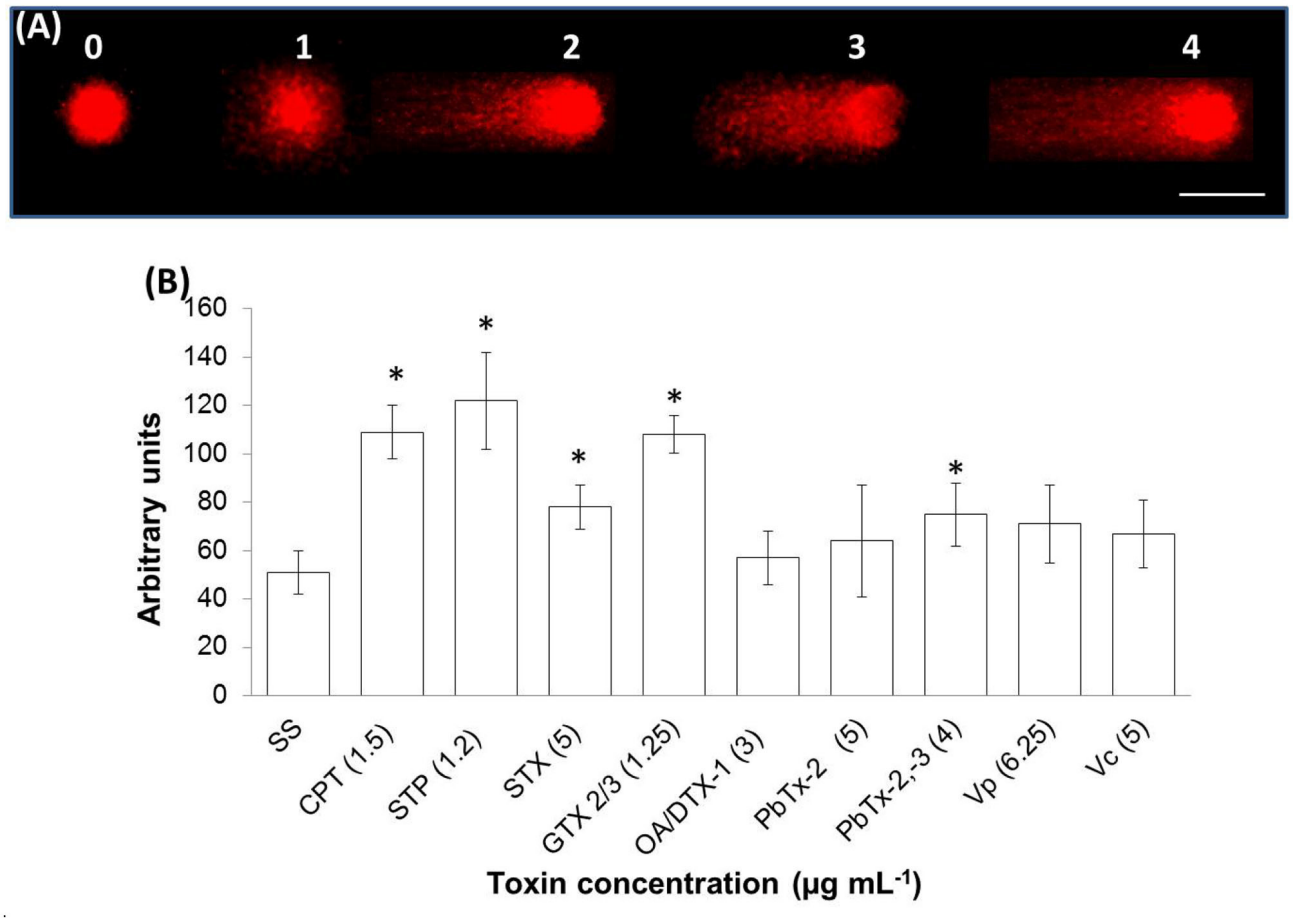
*In vitro* DNA double-strand breakage in hemocytes exposed to apoptosis inducers or marine toxins for 4 h at 25°C. **(A)** Image of hemocyte nuclei with different DNA damage grades, assessed by neutral comet assay, stained red with propidium iodide. DNA damage categories: undamaged, low damaged, medium damage, high damage, and complete damage, using a scale of 0–4, respectively. Scale bar = 5 μm. **(B)** Frequency distribution of DNA damage in hemocytes. Data were obtained from 400 scored nuclei. Results are expressed as the mean ± standard deviation. *P < 0.05. SS, saline solution; CPT, camptothecin; STP, staurosporine; STX, saxitoxin; GTX, gonyautoxin; OA, okadaic acid; DTX, dynophysistoxin; PbTx, brevetoxin; Vp, *Vibrio parahaemolyticus* extract; Vc, *V. campbellii* extract.

**Figure 4:**
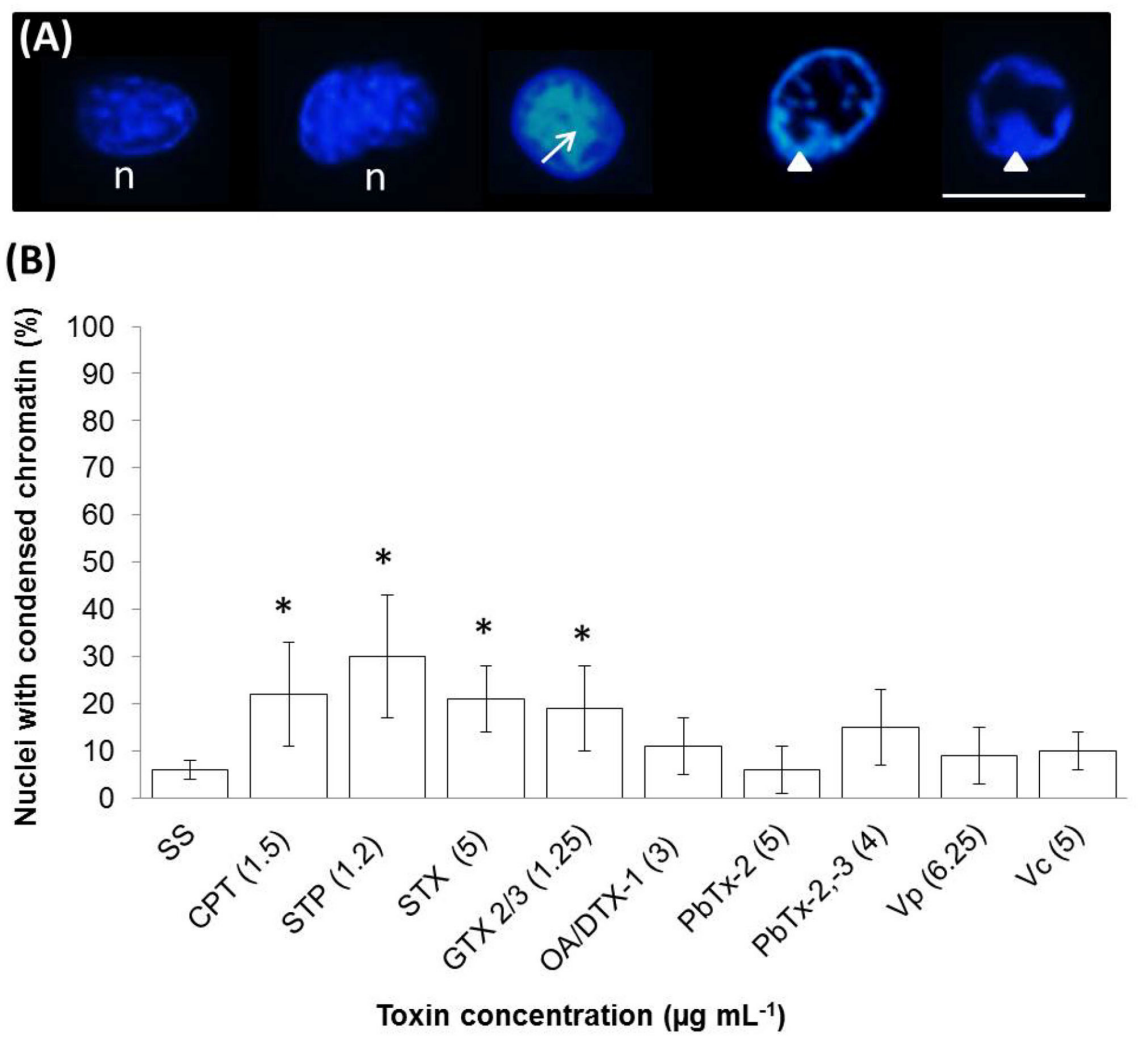
*In vitro* chromatin condensation in nuclei of hemocytes exposed to apoptosis inducers or marine toxins for 4 h at 25°C. **(A)** DAPI staining (blue) of representative nuclei: observe the normal nuclei (n), hyperchromasia (arrow) characteristic of condensed chromatin, and nuclei with condensation of chromatin in the periphery (arrow head). **(B)** Percentages of the nuclei with condensed chromatin. In each sample, at least 100 nuclei were counted. Scale bar = 5 μm. Results are expressed as the mean ± standard deviation. *P < 0.05. SS, saline solution; CPT, camptothecin; STP, staurosporine; STX, saxitoxin; GTX, gonyautoxin; OA, okadaic acid; DTX, dynophysistoxin; PbTx, brevetoxin; Vp, *Vibrio parahaemolyticus* extract; Vc, *V. campbellii* extract.

**Figure 5:**
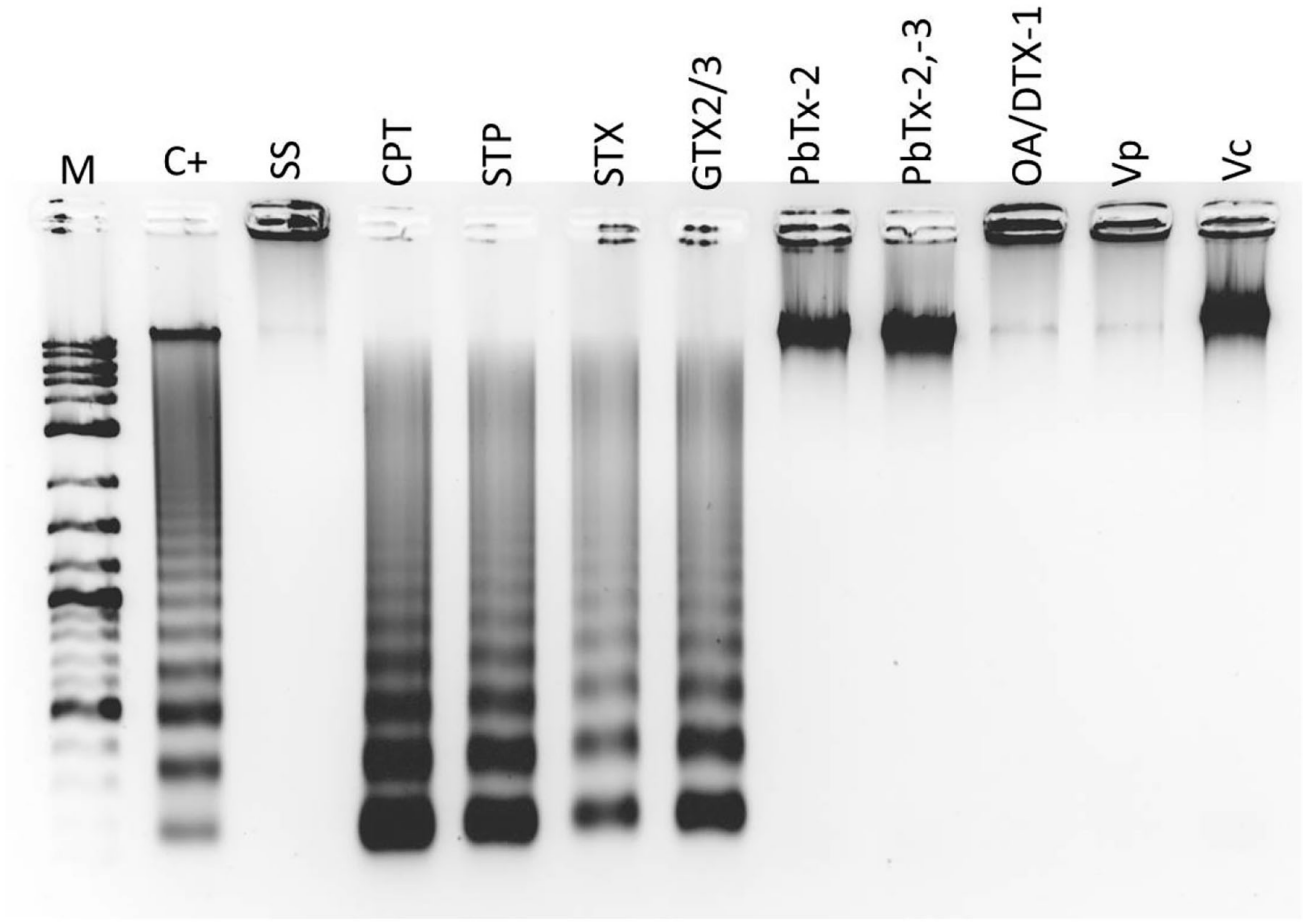
*In vitro* DNA ladder in nuclei of hemocytes exposed to apoptosis inducers or marine toxins for 4 h at 25°C. Electrophoresis was performed on 2% agarose gel. M, kb marker; C+, Positive control (DNA from apoptotic U937 cells); SS, Saline solution; CPT, camptothecin; STP, staurosporine; STX, saxitoxin; GTX, gonyautoxin; OA, okadaic acid; DTX, dynophysistoxin; PbTx, brevetoxin; Vp, *Vibrio parahaemolyticus* extract; Vc, *V. campbellii* extract.

A late stage of PCD pathway is the breakage of double-stranded DNA. To confirm that marine toxins induce PCD and analyze bivalves' and vertebrates' PCD pathway similitudes, we measure marine toxins' effect in double-stranded DNA breakage. Hemocytes were incubated with toxins as described above and analyzed by neutral single-cell gel electrophoresis (comet) assay. Broken DNA migrates electrophoretically faster than complete DNA material, a phenomenon that results in the formation of a “tail” susceptible to stain a measure in fluorescence images. More DNA breakage produces longer tails susceptible to categorize in the classes shown in Figure 3A. The statistical analysis shown in Figure 3B indicates that hemocytes incubated with CTP, STP, STX, GTX 2/3, and PbTx-2,-3 developed higher DNA breakage than the observed in control hemocytes exposed to sterile saline solution NaCl 0.9% (*P* < 0.05). Besides DNA breakage, PCD cells develop nuclear hyperchromasia and chromatin condensation, observable by fluorescence. We stained hemocytes incubated in the absence or presence of marine toxins with DAPI, as indicated before and measured the number of hyperchromatic and peripheral chromatin condensation nuclei following the characteristics illustrated in Figure 4A. The quantitative analysis presented in Figure 4B proves that CTP, STP, STX, and GTX 2/3, induced hyperchromasia and chromatin condensation in hemocytes (*P* < 0.05). Analyses of DNA breakage, nuclear hyperchromasia, and chromatin condensation confirm that CTP, STP, STX, and GTX 2/3 induce PCD and that bivalve mollusk and vertebrates' PCD signaling pathways are fundamentally similar. We also tested the nuclear DNA fragmentation, and Figure 5 shows DNA laddering visualized in a 2% agarose gel. We used as a positive control (C+), DNA from U937 apoptotic cells provided by the kit. A clear DNA-ladder pattern is visible when hemocytes are treated with CPT, STP, STX, and epimers GTX 2/3. None oligonucleosomal fragments were observed in the rest of the samples.

### Caspase Gene Expression

**Figure 6:**
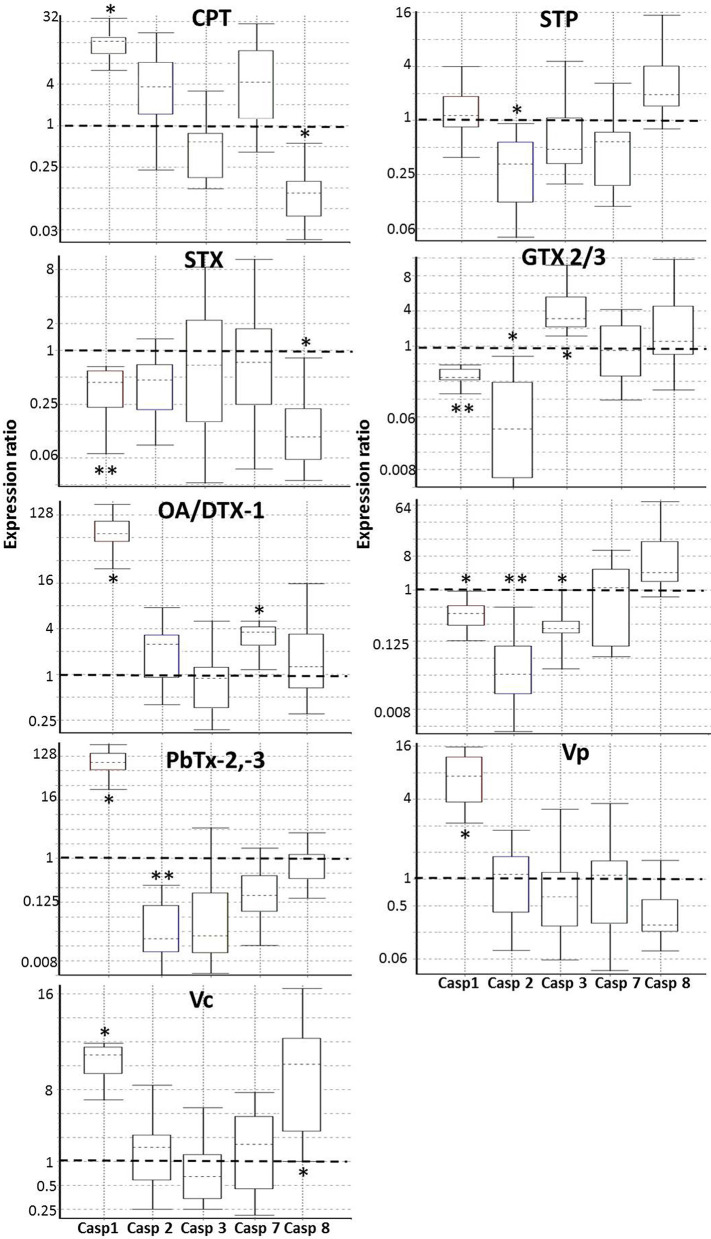
Real-time PCR (qRT-PCR) of caspases (Casp) in hemocytes exposed to in hemocytes exposed to apoptosis inducers or marine toxins for 4 h at 25°C. Whisker-box plots of the relative expressions calculated by the REST 2009 Software of the caspase-1, caspase-2, caspase-3, caspase-7, and caspase-8 genes. Boxes represent the interquartile range, or the middle 50% of observations. The dotted line inside the box represents the median gene expression. Whiskers represent the minimum and maximum observations. The proportions over 1 indicate genes that increase expression, while proportions <1 indicate genes decrease in expression. *P < 0.05; **P < 0.01. Casp, Caspase; CPT, Camptothecin; STP, Staurosporine; STX, Saxitoxin; GTX, Gonyautoxin; OA, Okadaic acid; DTX, Dynophysistoxin; PbTx, Brevetoxin; Vp, *Vibrio parahaemolyticus* extract; Vc, *V. campbellii* extract.

Once confirmed that some marine toxins induce PCD, we investigated what kind of PCD these substances trigger. Based on what is known in vertebrates, we hypothesized that marine toxins induce either apoptosis, a process characterized by the activation of caspases−2,−3,−7, or−8, or pyroptosis-like, identified by the increment in the expression of the proinflamatory caspase-1. We measured the amount of mRNA of these caspases by RT-qPCR, in the absence or presence of marine toxins and vertebrates' apoptosis inducers, as described before (Figure 6 and Table 3). Caspase-1 mRNA underwent the most drastic downregulation in hemocytes treated with STX, GTX2/3, and PbTx-2, and a significant upregulation in the cells treated with CPT, AO/DTX1, PbTx-2,−3, Vc, and Vp. Caspase-2 mRNA decreased in hemocytes exposed to STP, GTX2/3, PbTx-2, PbTx-2,-3, while caspase-3 mRNA increased under GTX2/3 treatment and decreased with PbTx-2 incubation. Caspase-7 mRNA increased in hemocytes exposed to AO/DTX-1 and, finally, caspase-8 mRNA decreased in hemocytes incubated with CPT, and STX, and increased in hemocytes exposed to Vc crude extract. Table 3 is a matrix that summarizes the graphical data of RT-qPCR, representing the value of significance (*P*) for each sample with a colorimetric scale (green, up-regulated; red down-regulated; yellow, no-differences).

**Table 3:** *P*-value of relative expression of caspase transcripts in hemocytes of *Crassostrea gigas* exposed to apoptosis inducers or marine toxins.

**Toxin**	**caspase-1**	**caspase-2**	**caspase-3**	**caspase-7**	**caspase-8**
CPT	0.025	0.051	0.968	0.083	0.030
STP	0.326	0.013	0.142	0.146	0.223
STX	0.001	0.055	0.396	0.586	0.015
GTX2/3	0.000	0.033	0.047	0.534	0.158
OA/DTX-1	0.028	0.074	0.486	0.014	0.093
PbTx2	0.026	0.000	0.030	0.437	0.200
PbTx2/3	0.019	0.000	0.088	0.308	0.574
Vp	0.020	0.968	0.390	0.800	0.105
Vc	0.021	0.173	0.459	0.273	0.014

*CPT, Camptothecin; STP, Staurosporine; STX, Saxitoxin; GTX, Gonyautoxin; OA, Okadaic acid; DTX, Dynophysistoxin; PbTx, Brevetoxin; Vp, Vibrio parahaemolyticus extract; Vc, V. campbellii extract. Red = down regulated; Green = up-regulated; Yellow = No differences comparing with control*.

## Discussion

The PCD concept applies broadly to several intracellular pathways involved in cell's self-destruction (21). PCD exhibit unique morphological characteristics and energy-dependent biochemical mechanisms, and occurs when a cell is damaged, infected, senescent, or otherwise of little use to the animal (20–24). PCD participates in the immune system (79–81), and marine toxins modulated mollusk's immune response (9, 25, 28, 29, 31, 46–52, 55, 56, 58–62, 82–87). Hemocytes are the first line of defense in bivalve mollusks immune system, and here we evaluated how marine toxins modulate PCD in hemocytes from *C. gigas*. The fact that control hemocytes exhibit high PCD level indicates that this process is vital for molluscan immunity (25, 26, 88–90). Here we show that marine toxins increase hemocyte's cell mortality with PCD phenotype.

We first analyzed the cell death characteristics induced by CPT and STP, two *bona fide* PCD inducers. CPT is a potent inhibitor of topoisomerase I extracted from the Chinese tree *Camptotheca acuminata* (91). STP is a non-selective protein kinase inhibitor obtained from the bacteria *Streptomyces staurospores* (92). Both substances induce apoptosis *in vitro* in various cell types and therefore they are standard positive controls in bioassays (92–96). Both substances caused high mortality in *C. gigas* hemocytes and helped to describe how death is carried out in bivalve hemocytes, as previously shown for *Nodipecten subnodosus* (58). Like STP and CPT, hemocyte death was evident with paralyzing toxins (STX and epimers GTX2/3), and to a lesser extent, with the mixture of brevetoxins (PbTx-2,-3). The inducers and these marine toxins, showed a marked translocation of PS to the extracellular leaflet and breakage of double-stranded DNA. Early PS exposure is closely associated with plasma membrane rupture during PCD for attracting engulfing cells (43, 45, 97–99). We observed many cells expressing annexin V and PI labels and considered them to be in late PCD or result from secondary necrosis, but we thought them for the analysis only as dead cells, and it was not the study's object of this work. Recent studies showed that mammalian cell lines undergoing programmed necrosis (necroptosis), as well as necrotic cells in the nematode *Caenorhabditis elegans*, translocates PS to their outer surfaces before cell lysis to recruit phagocytes (100, 101). Chromatin condensation and DNA fragmentation are important criteria to identify PCD in terminal stages. We observed both characteristics consistently in hemocytes treated with STP, CPT, STX, and epimers GTX2/3, which induced a clear death ladder pattern. During apoptosis and pyroptosis, cells undergo chromatin condensation and DNA fragmentation, but only in apoptosis the nucleus breaks into multiple chromatin bodies, in a process called nucleosomal fragmentation, while remains intact in pyroptosis (39–44). In previous work we observed that paralyzing toxins, epimers GTX2/3, provokes nucleosomal fragmentation and apoptosis in *N. subnodosus* (58).

Caspases are a family of cysteine proteases, which play an essential role in apoptosis and inflammation, distinguishing between many cell death possibilities. Caspases involved in apoptosis are human caspase -2, -3, -6, -7, -8, -9, and -10; those participating primarily in pyroptosis are human caspase -1, -4, -5, -13, and -14, as well as murine caspase -11 and -12 (21, 28, 32, 102). *C*. *gigas* expresses executer caspases -1, -3, and -7 and initiator caspases -2 and -8 (30, 37, 55, 103, 104). Given that mRNA increase generally indicates the synthesis of a particular gene product (105), we resorted to use mRNA quantitation to measure caspase expression. CPT induced upregulation of the pro-inflammatory caspase-1 and down regulation of caspase 8. Caspase-1 is a cysteine protease that cleaves and activates the pro-forms of host inflammatory cytokines, IL-1β, and IL-18 (106). On vertebrates, caspase-1 acts in pyroptosis, a pathway of host cell death stimulated by a range of microbial infections and non-infectious stimuli, associated with plasma membrane rupture and release of pro-inflammatory intracellular content (35, 38, 107, 108). Therefore, we show here that CPT could induces pyroptosis-like in *C*. *gigas* hemocytes, in addition to cause apoptosis. Caspase-1 mediated pyroptosis has been observed in other invertebrates such as sea cucumbers (109) and crustaceans (110). *C. gigas* hemocytes express cytoplasmic and nuclear caspase-1, capable to induce cell death (37, 111). In oysters, caspase-1 is a homolog of executioner caspase-3/7, which can activate itself, bind to other caspases and lipopolysaccharides (111). On the other hand, STP induces caspase-2 down-regulation in hemocytes of *C. gigas*. Caspase-2 keeps a high similarity among animal species (37, 51) and the pathogen-associated molecular patterns (PAMPs) provoke its downregulation in *Mytilus edulis* mussel (51). Nevertheless, STP provoked apoptosis in bivalve hemocytes of *N. subnodosus* dependent of caspases (58), which indicates that the type of PCD that STP induces depends on the species studied. The low level of variation of caspases mRNA induced by CPT and STP seems to be sufficient to induce PCD in *C. gigas*, either by apoptosis or pyroptosis-like, given that both substances induced clear morphological alterations such as the breakage of double-stranded DNA, induction of the characteristic DNA ladder pattern, and the translocation of PS to the extracellular leaflet of the plasma membrane.

Paralyzing shellfish toxins (PST) are tricyclic tetrahydropurine derivatives with potent hydrophilic neurotoxic activity. In vertebrates, PSTs inhibit the voltage gated-sodium channel with high affinity and, thus blocking action potentials in excitable membranes of neurons and muscles (112). PSTs are present in some genera of dinoflagellates such as *Alexandrium* sp., *Pyrodinium bahamense, Gymnodinium catenatum, Centrodinium punctatum* and cyanobacteria (113–116). There are more than 57 analogs of these toxins that differ in their toxicity (117), and has been stated that PSTs provoke apoptosis *in vivo* e *in vitro* in bivalve mollusks (55, 58, 87). In *C. gigas* hemocytes the epimers GTX2/3 increased expression of executioner caspase-3 and with STX a down-regulated caspase-8 was observed. Caspase-3 and caspase-8 have been previously identified in *Crassostrea* sp. (30, 37, 100, 118). These results could indicate that hemocytes are in a late stage of apoptosis, consistent with the observed oligonucleosomal fragmentation. These results further corroborate that marine toxin induce PCD by apoptosis in hemocytes of *C. gigas*. When STX is injected in the mussel *M. chilensis*, hemocytes involve numerous pattern recognition receptors (PRRs) that, subsequently, trigger a cellular response apoptotic or autophagic death (87). When epimers GTX2/3 are exposed with the hemocytes of the pectinid *N. subnodosus*, the induction of apoptosis is linked directly to caspases (58). When *C. gigas* fed with the PST producer *A. catenella* there was a significant increase of the number of hemocytes in apoptosis after 29 h of exposure, with overexpression of two caspase executor genes (caspase-3 and caspase-7) (55). STX and GTX 2/3 also showed the down-regulation of caspase-1 in *C. gigas*, which indicates that PST inhibits pyroptosis-like processes, similar to what happens with other pathogens, conferring some ability to persist and cause disease in the host (35). On the other hand, this inhibition of caspase-1 could permit hemocytes perform apoptosis in an orderly manner, thus avoiding the inflammatory damage.

Brevetoxins (PbTxs) represent a group of polyether compounds that bind to and stimulate sodium flux through voltage-gated sodium channels in nerve and muscle, leading to uncontrolled sodium influx into the cell (119). PbTxs are produced by the marine dinoflagellate *Karenia brevis* (119, 120). The mixture of PbTx-2,-3 triggered DNA alterations, PS translocation, and mortality in *C. gigas* hemocytes. PS translocation and chromosomal DNA cleavage are observed in apoptosis and pyroptosis (35, 42–45). After hemocyte exposure to a mixture of these toxins, we did not observe the DNA ladder characteristic of apoptosis, and together with the up-regulation of caspase-1 and the absence of nuclear disintegration, suggest a PCD by a pyroptosis-like mechanism. In human lymphocytes exposed to PbTx-2,-3, results indicate a high mortality rate and extensive genotoxic damage with both toxins (121). On the contrary, when we exposed hemocytes of *C. gigas* to PbTx-2, caspase-1, initiator caspase-2, and effector caspase-3 expression decreased, and cell death was low, in agreement with the experiments carried out by Mello et al. (52), where at 4 h, high viability was observed with the same toxin in *C. gigas*. They concluded that this viability correlated with the activation of detoxification and stress genes CYP356A1, FABP and Hsp70, but not with immune or to antioxidant ones BPI, IL-17, EcSOD, Prx6, GPx, and SOD.

Diarrheic shellfish toxins (DST) are heat-stable polyether and lipophilic compounds isolated from various species of dinoflagellates, mainly of the genus *Dynophysis* and *Prorocentrum* (114). Among these toxins, okadaic acid (OA) and its derivatives named dinophysistoxins (DTX1-10) are the best known. These compounds inhibit protein phosphatase-1 and -2A *in vitro*, provoking inflammation of the intestinal tract and diarrhea in humans (122, 123). They are also tumor promoters in animal test systems (124, 125). OA and its analogs DTXs did not seem to cause the same harmful effects in bivalve hemocytes as in other studied vertebrate cell lines, where OA induced cytotoxicity and apoptosis (126–136). Our analyses with the mixture of OA and DTX-1, showed no evident cytotoxic effects *in vitro* in hemocytes of *C. gigas*. It has been demonstrated in *C. gigas* that ingestion of the strain of *P. lima* cells that produces OA and DTX1, provokes a clear mRNA modulation expression of the genes involved in cell cycle regulation and immune system (137). Several authors have pointed out that the low percentage of dead hemocytes in bivalve mollusks and apoptotic processes, seem to indicate *in vivo* and *in vitro*, that these organisms have protective mechanisms and resistance against harmful effects of OA and/or DTX-1, that involves OA's storage into the lysosomal system (50, 56, 60, 138). Despite low hemocyte death in our work, there was a significant increase in caspase-1 and caspase-7 when hemocytes were exposed to OA/DTX1. Caspase-1 can process proteolytically the apoptotic effector caspase-7, and both can activate simultaneously (36, 139). There are vertebrate cell types that express caspase-1 but do not undergo PCD (140). It has been described that the low level of cell death when exposed to OA indicates protective mechanisms by the presence of caspase inhibitors, which can inhibit PCD pathways (141).

Bacteria of the genus *Vibrio* sp. produce many pathogenic factors, including enterotoxins, hemolysins, and cytotoxins. *V. campbellii* and *V. parahaemolyticus* are extensively studied species because they are the causative agent of the often lethal effects in aquaculture organisms, such as fish, bivalves, and crustacean (142–145). In this study, hemocytes were exposed to the crude protein extract of *V. parahaemolyticus*, and *V. campbellii*, two bacteria that had shown high virulence against shrimp postlarvae, and were isolated from shrimp and oyster, respectively (146). The crude extracts of these bacteria showed hemolytic activity in human erythrocytes (147). Still, we did not find a cytotoxic effect at 4 h post challenged. We previously identified genes related to some protein toxins as part of this virulence, and their genomes showed many pathogenic factors such as hemolysin, enterotoxins, cytotoxins, proteases, siderophores, adhesive factors, and hemagglutinin (148). Some studies with mollusks have demonstrated the role of caspase-8 in anti-bacterial response (51, 118, 149–151), and it has been proved in *Haliotis discus discus* and *C. hongkongensis* that caspase-8 mRNA expression in hemocytes was significantly up-regulated by exposure to *Vibrio* species (118, 150). Similar results we obtained with proteinaceous extract from *V. campbellii* in *C. gigas* hemocytes. Caspase-8 was cloned and characterized in *C. hongkongensis* and *C. gigas* and showed that it is ubiquitously expressed in oysters, suggesting a role in apoptosis (100, 118). Likewise, *Vibrio* extracts showed the over-expression of caspase-1 in hemocytes from *C. gigas*. It has been recognized that moderate activity levels of caspase-1 stimulate cell host's survival responses, modulate intracellular growth of bacteria, and promote inflammatory cytokines production. When caspase-1 passes a threshold level, the cell undergoes PCD by pyroptosis, characterized by plasma-membrane pore formation, which leads to cell lysis and release of pro-inflammatory intracellular contents (35, 42, 152, 153). Pore-forming toxins are the most common *Vibrio* cytotoxic proteins, are required for virulence, and have been implicated in pyroptosis cell death (154, 155). Also, several studies have identified novel roles for caspase-8 in modulating IL-1β, inflammation, and caspase-1 processing, in response on the stimulus or stimuli that initiate the signaling cascade (156–163). In *C. gigas* a pyroptosis-like PCD could be playing a role in bacteria clearance, by removing intracellular replication niches and enhancing the host's immune responses. Also it has been documented that caspase-1 activation fails to trigger pyroptosis in many vertebrate cell lines in response to bacterial pore-forming toxins, which in turn promote cell survival upon toxin challenge possibly by facilitating membrane repair (164), a similar scenario could be relevant for infectious with *Vibrio* extracts in *C. gigas* hemocytes. It is controversial whether pyroptosis, which can also be triggered by non-bacterial pathological stimuli, truly represents a cell modality in bivalve hemocytes or whether it constitutes a special case of apoptosis or necrosis, but the activation of caspase-1 in cells could has unquestionable pathophysiological implications.

Finally, we do not rule out the importance of different cell death types that could be causing misbalance of hemocytes, such as necrosis, necroptosis, autophagy, pyroptosis, pironecrosis, etc. The marine non-proteinaceous STX, GTX2/3, and PbTx-2,-3 trigger signaling pathways that promote apoptotic cell death, as the apoptotic inducers CPT and STP, but PbTx-2,-3 did not show oligonucleosomal fragmentation. Vp and Vc extracts, OA/DTX-1, and PbTx-2 increased pro-inflammatory caspase-1 indicative of signaling pathways leading to PCD by a pyroptosis-like process; still, protective mechanisms could influence in the low cell death observed. The results presented illustrate the complexity of the hemocyte response to marine toxins. Nevertheless, they are consistent with the role of PCD to preserve a healthy and balanced immunity, keeping hemocytes at normal levels for the systematic and regulated dismantling elimination of damaged cells in *C. gigas*.

## Data Availability Statement

The datasets generated for this study are available on request to the corresponding author.

## Author Contributions

NE, EN-V, AP, FA, and RC contributed to the conception and design of the study. NE, EN-V, AP, and LG-V developed the experiments and performed statistical analysis. NE, EN-V, AP, LG-V, and RC analyzed the data as well as wrote the paper. All authors contributed to revising the manuscript, reading, and approving the final version.

## Conflict of Interest

The authors declare that the research was conducted in the absence of any commercial or financial relationships that could be construed as a potential conflict of interest.
