# Supplementary material for: In vitro Evaluation of Programmed Cell Death in the Immune System of Pacific Oyster Crassostrea gigas by the Effect of Marine Toxins
Source: Front Immunol. 2021 Apr 1;12:634497. doi: 10.3389/fimmu.2021.634497 (PMC8047078; doi:10.3389/fimmu.2021.634497)
Supplement: Supplementary file 1 [file Data_Sheet_1.docx]

**Table S1**. PCR and qPCR primers sequences used in this study

| **GENE** | **PRIMER** | **PRODUCT**  **(bp)** |
| --- | --- | --- |
| **End point PCR** | | |
| 16S  RNAr^1^ | F 5´ AGAGTTTGATCMTGGCTCAG 3´  R 5´ ACCTTGTTACGACTT 3´ | 1500 |
|  |  |  |
| VA-p^2^ | F 5´ AGACACGGTCCAGACTCCTAC 3´  R 5´ AGGGTATCTAATCCTGTTTGCT 3´ | 474 |
|  |  |  |
| VPM^3^ | F 5´CAGCTACCGAAACAGACGCTA  R 5´TCCTATCGAGGACTCTCTCAAC | 675 |
|  |  |  |
| ToxR1^4^ | F 5´ GTCTTCTGACGCAATCGTTG 3´  R 5´ ATACGAGTGGTTGCTGTCATG 3´ | 368 |
|  |  |  |
| ToxR2^5^ | F 5´ GAACCAGAAGCGCCAGTAGT 3´  R 5´ GCATGGTGCTTAACGTAGCG 3´ | 257 |
|  |  |  |
| Tlh^6^ | F 5´ AAAGCGGATTATGCAGAAGCACTG 3´  R 5´ GCTACTTTCTAGCATTTTCTCTGC 3´ | 450 |
|  |  |  |
| Tdh^7^ | F 5´GTAAAGGTCTCTGACTTTTGGAC 3´  R 5´ TGGAATAGAACCTTCATCTTCACC 3´ | 269 |
|  |  |  |
| Trh^8^ | F 5´TTGGCTTCGATATTTTCAGTATCT  R 5´CATAACAAACATATGCCCATTTCCG | 500 |
|  |  |  |
| OmpW^9^ | F 5´ CACCAAGAAGGTGACTTTATTGTG 3´  R 5´ GAACTTATAACCACCCGCG 3´ | 588 |
|  |  |  |
| CtxAB^10^ | F 5 ’CTCAGACGGGATTTGTTAGGCACG ’3  R 5’ TCTATCTCTGTAGCCCCTATTAC G ’3 | 301 |
|  |  |  |
| PirA^11^ | F 5´TGACTATTCTCACGATTGGACTG  R 5´CACGACTAGCGCCATTGTTA | 284 |
|  |  |  |
| PirB^11^ | F 5´TGATGAAGTGATGGGTGCTC  R 5´TGTAAGCGCCGTTTAACTCA | 392 |
|  |  |  |
| **qPCR** | | |
| Casp 1^12^ | F 5´ CTGAACGAGCGGAATGGCA 3´  R 5´ CCTGACCTTTCTGTAGTGTA 3´ | 133 |
|  |  |  |
| Casp 2^13^ | F 5´ACAGGGGAAATACTGAAGGAC 3´  R 5´ AGCTACAGCTGTCAGAAACC 5´ | 165 |
|  |  |  |
| Casp 3^12^ | F 5´ CGGGAAATTACGGGGAGTTG 3´  R 5´TCTTCGGAGGATACAGAGGG 3´ | 139 |
|  |  |  |
| Casp 7^13^ | F 5´ ATTGGACCACAGAGACAACG 3’  R 5´ TGTTGCCTTTGAAGGGCTCC 3’ | 135 |
|  |  |  |
| Casp 8^14^ | F 5´ CAGGGAAGCTGGTTCGTCAA 3´  R 5´ CCCTCCAGTCGGGCGTTA 3´ | 125 |
|  |  |  |
| RL 7^15^ | F 5´ TCCCAAGCCAAGGAAGGTTATGC 3´  R 5´ CAAAGCGTCCGAGGTGTTTCTCAA 3 | 242 |
|  |  |  |
| RL 36^15^ | F 5´ CATAGAACCATTCCTCTGAAAGCTG 3´  R 5´ CGTAGGGAGCAAATCCTGTGA 3´ | 124 |

VPM, *Vibrio parahaemolyticus* metalloprotease; ToxR, transmembrane DNA-binding protein; Tlh, Thermolabil hemolysin; Tdh, Thermostable direct hemolysin; Trh, thermostable direct hemolysin-related gene; OmpW, Outer membrane protein; CtxAB, Cholera toxin; Casp, caspase; RL, Ribosomal protein.

*References for primer sequences sourced from the literature are provided below.

1. Lane DJ. “16S/23S rRNA sequencing”. In: Stackebrandt E, Goodfellow M, editors. Nucleic acid techniques in bacterial systematics. Chichester, England: John Wiley and Sons (1991)**.** p. 115–147.

2. Xu H, He LH, Lv S, Gong O, Li SL. Establishment of universal loop-mediated isothermal amplification method (LAMP) for rapid detection of pathogenic *Vibrio* spp. in aquatic organisms. *African J Microbiol Res.* (2012) 6(14):3447–3454. doi: 10.5897/AJMR11.1417

3. Luan XY, Chen JX, Zhang XH, Jia JT, Sun FR, Li Y. Comparison of different primers for rapid detection of *Vibrio parahaemolyticus* using the polymerase chain reaction. *Lett. Applied Microbiol.* (2007) 44(3):242–247. doi: 10.1111/j.1472-765x.2006.02074.x

4. Kim YB, Okuda J, Matsumoto C, Takahashi N, Hashimoto S, Nishibuchi M. Identification of *Vibrio parahaemolyticus* strains at the species level by PCR targeted to the toxR gene. *J Clin Microbiol.* (1999) 37:1173–1177. doi: 10.1128/JCM.37.4.1173-1177.1999

5. Wang D, Fang Z, Xie C, Liu Y. Construction of method for rapid detection of *Vibrio* *parahaemolyticus* using the quantitative real-time PCR based on the ToxR gene. *Adv J Food Sci Tech.* (2013) 5(8):1022–1030. doi: 10.19026/ajfst.5.3200

6. Taniguchi H, Hirano H, Kubomura S, Higashi K, Mizuguchi Y. Comparison of the nucleotide sequences of the genes for the thermostable direct hemolysin and the thermolabile hemolysin from *Vibrio parahaemolyticus*. *Microb Pathog.* (1986) 1(5):425–432. doi: 10.1016/0882-4010(86)90004-5

7. Nishibuchi M, Kaper JB. Thermostable direct hemolysin gene of *Vibrio parahaemolyticus*: a virulence gene acquired by a marine bacterium. *Infect Immun* (1995) 63(6):2093–2099. doi: 10.1128/IAI.63.6.2093-2099.1995

8. Bej AK, Patterson DP, Brasher CW, Vickery MCL, Jones DD, Kaysner CA. Detection of total and hemolysin-producing *Vibrio parahaemolyticus* in shellfish using multiplex PCR amplification of tl, tdh and trh. *J Microbiol Methods.* (1999) 36(3):215–225. doi: 10.1016/s0167-7012(99)00037-8

9. Nandi B, Nandy RK, Mukhopadhyay S, Nair GB, Shimada T, Ghose AC. Rapid method for species-specific identification of *Vibrio cholerae* using primers targeted to the gene of outer membrane protein OmpW. *J Clin Microbiol.* (2000) 38:4145–4151. doi: 1010.1128/jcm.38.11.4145-4151.2000

10. Thompson CC, Freitas FS, Marin MA, Fonseca EL, Okeke IN, Vicente ACP. *Vibrio cholerae* O1 lineages driving cholera outbreaks during seventh cholera pandemic in Ghana. *Infect Genet Evol.* (2011) 11(8):1951–1956. doi: 10.1016/j.meegid.2011.08.020

11. Han J, Tang K, Tran L, Lightner D. Photorhabdus insect-related (Pir) toxin-like genes in a plasmid of *Vibrio parahaemolyticus,* the causative agent of acute hepatopancreatic necrosis disease (AHPND) of shrimp. *Dis Aquat Organ.* (2015) 113(1):33–40. doi: 10.3354/dao02830

12. Qu T, Huang B, Zhang L, Li L, Xu F, Huang W, Li C, Yishuan D, Zhang G. Identification and functional characterization of two executioner caspases in *Crassostrea gigas*. *PLoS ONE*. (2014) 9(2):e89040. doi: 10.1371/journal.pone.0089040

13. Medhioub W, Ramondenc S, Vanhove A, Vergnes A, Masseret E, Savar V, Amzil Z, Laabir M, Rolland J. Exposure to the neurotoxic dinoflagellate, *Alexandrium catenella,* induces apoptosis of the hemocytes of the oyster, *Crassostrea gigas*. *Mar Drugs.* (2013) 11(12):4799–4814. doi: 10.3390/md11124799

14. Li C, Qu T, Huang B, Ji P, Huang W, Que H, Li L, Zhang G. Cloning and characterization of a novel caspase-8-like gene in *Crassostrea gigas*. *Fish Shellfish Immun*. (2015) 46(2):486–492. doi: 10.1016/j.fsi.2015.06.035

15. Du Y, Zhang L, Xu F, Huang B, Zhang G, Li L. Validation of housekeeping genes as internal controls for studying gene expression during Pacific oyster (*Crassostrea gigas*) development by quantitative real-time PCR. *Fish Shellfish Immunol.* (2013) 34(3):939–945. doi: 10.1016/j.fsi.2012.12.007

**Table S2**. *Vibrio campbellii*, and *V. parahaemolyticus* amplified genes

| Strain/ Genes | 16s | Vap | VPM | ToxR | Tlh | Tdh | Trh | OmpW | CtxAB | PirA | PirB |
| --- | --- | --- | --- | --- | --- | --- | --- | --- | --- | --- | --- |
| *Vibrio campbellii* | + | + | - | + | - | - | - | + | - | - | - |
| *Vibrio parahaemolyticus* | + | + | + | + | + | + | - | + | - | + | + |

+, positive; -, negative.

VPM, *Vibrio parahaemolyticus* metalloprotease; ToxR, transmembrane DNA-binding protein; Tlh, Thermolabil hemolysin; Tdh, Thermostable direct hemolysin; Trh, thermostable direct hemolysin-related gene; OmpW, Outer membrane protein; CtxAB, Cholera toxin.
